# Congenital Glioblastoma multiforme and eruptive disseminated Spitz nevi

**DOI:** 10.1186/s13052-016-0260-9

**Published:** 2016-05-14

**Authors:** Victor Desmond Mandel, Flavia Persechino, Alberto Berardi, Giovanni Ponti, Silvana Ciardo, Cecilia Rossi, Giovanni Pellacani, Francesca Farnetani

**Affiliations:** Department of Surgical, Medical, Dental and Morphological Sciences with Interest transplant, Oncological and Regenerative Medicine, Dermatology Unit, University of Modena and Reggio Emilia, via del Pozzo 71, Modena, 41124 Italy; Unità Operativa di Terapia Intensiva Neonatale, Dipartimento Integrato Materno-Infantile, Azienda Ospedaliero-Universitaria Policlinico, via del Pozzo 71, Modena, 41124 Italy; Department of Surgical, Medical, Dental and Morphological Sciences with Interest transplant, Oncological and Regenerative Medicine, Clinical Pathology Unit, University of Modena and Reggio Emilia, via del Pozzo 71, Modena, 41124 Italy; Terapia Intensiva Neonatale, Arcispedale Santa Maria Nuova, Istituto di Ricovero e Cura a Carattere Scientifico-IRCCS, viale Risorgimento 80, Reggio Emilia, 42123 Italy

**Keywords:** Congenital glioblastoma multiforme, Chemotherapy, Spitz nevi, Eruptive disseminated Spitz nevi, Reflectance confocal microscopy

## Abstract

**Background:**

Glioblastoma multiforme (GBM) is the deadliest malignant primary brain tumor in adults. GBM develops primarily in the cerebral hemispheres but can develop in other parts of the central nervous system. Its congenital variant is a very rare disease with few cases described in literature.

**Case presentation:**

We describe the case of a patient with congenital GBM who developed eruptive disseminated Spitz nevi (EDSN) after chemotherapy. Few cases of EDSN have been described in literature and this rare clinical variant, which occurs predominantly in adults, is characterized by multiple Spitz nevi in the trunk, buttocks, elbows and knees. There is no satisfactory treatment for EDSN and the best therapeutic choice is considered the clinical observation of melanocytic lesions.

**Conclusion:**

We recommend a close follow-up of these patients with clinical observation, dermoscopy and reflectance confocal microscopy (RCM). However, we suggest a surgical excision of the lesions suspected of being malignant.

## Background

Case reports and small case series of congenital glioblastoma multiforme (GBM) have suggested variable survivals, with the large majority of patients dying within weeks to months, but occasional patients survive for years [1]. The treatment is the result of an effective teamwork based on neurosurgery, chemotherapy and radiation therapy [1, 2]. We describe the case of a patient with congenital GBM who developed eruptive disseminated Spitz nevi (EDSN) after chemotherapy.

## Case Presentation

**Fig. 1:**
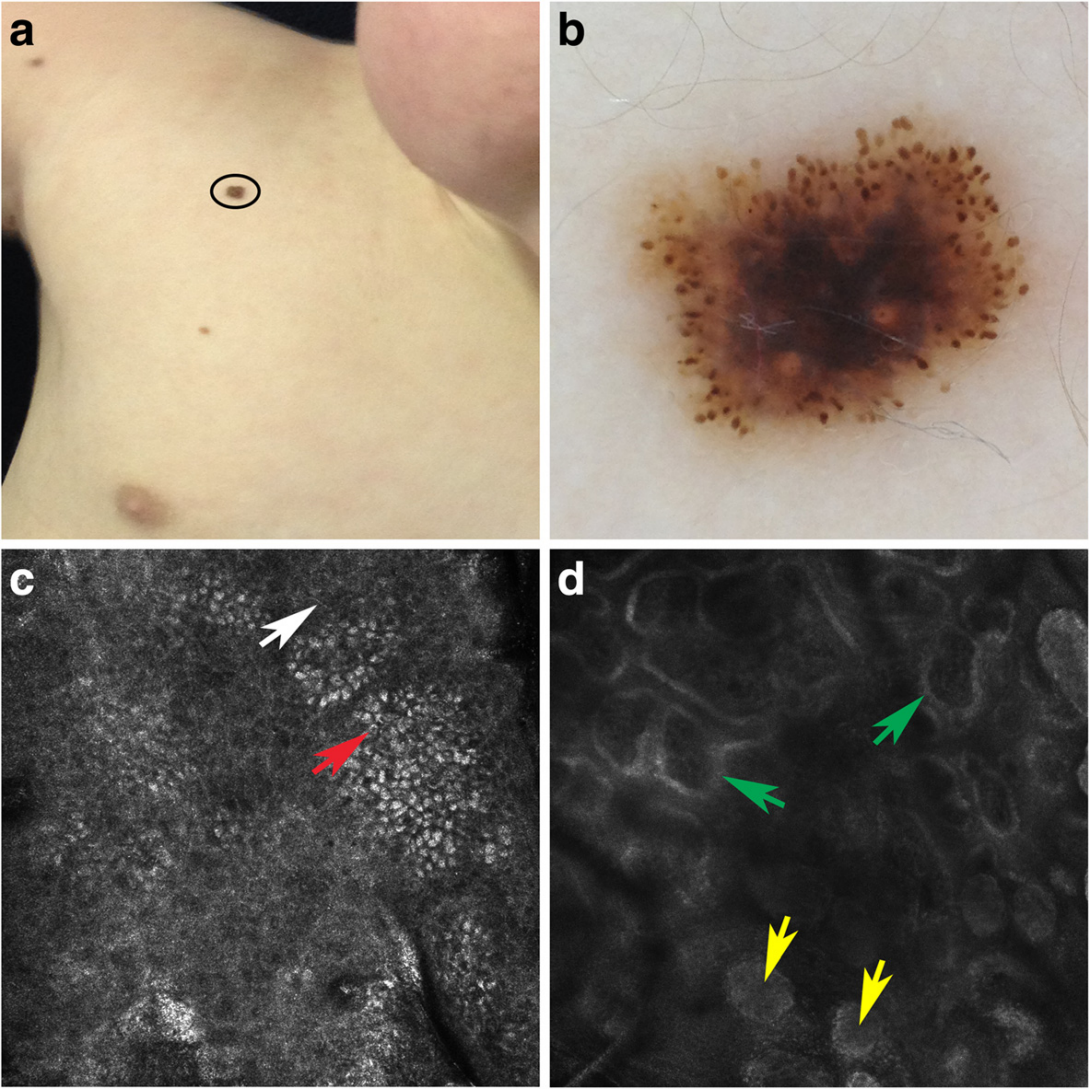
Spitz nevus localized on the shoulder (**a**). Dermoscopy showed a starbust pattern with multiple pigmented striations and large brown globules distributed symmetrically at the periphery of the lesion (**b**). RCM revealed in the epidermis regular cobblestone with rare spindled (*white* arrow) and atypical cells (*red* arrow) (**c**). Instead at the dermoepidermal junction and within the papillary dermis showed the typical dense regular nests (*yellow* arrows). Moreover, papillary dermis presented edged papillae (*green* arrows) (**d**)

**Fig. 2:**
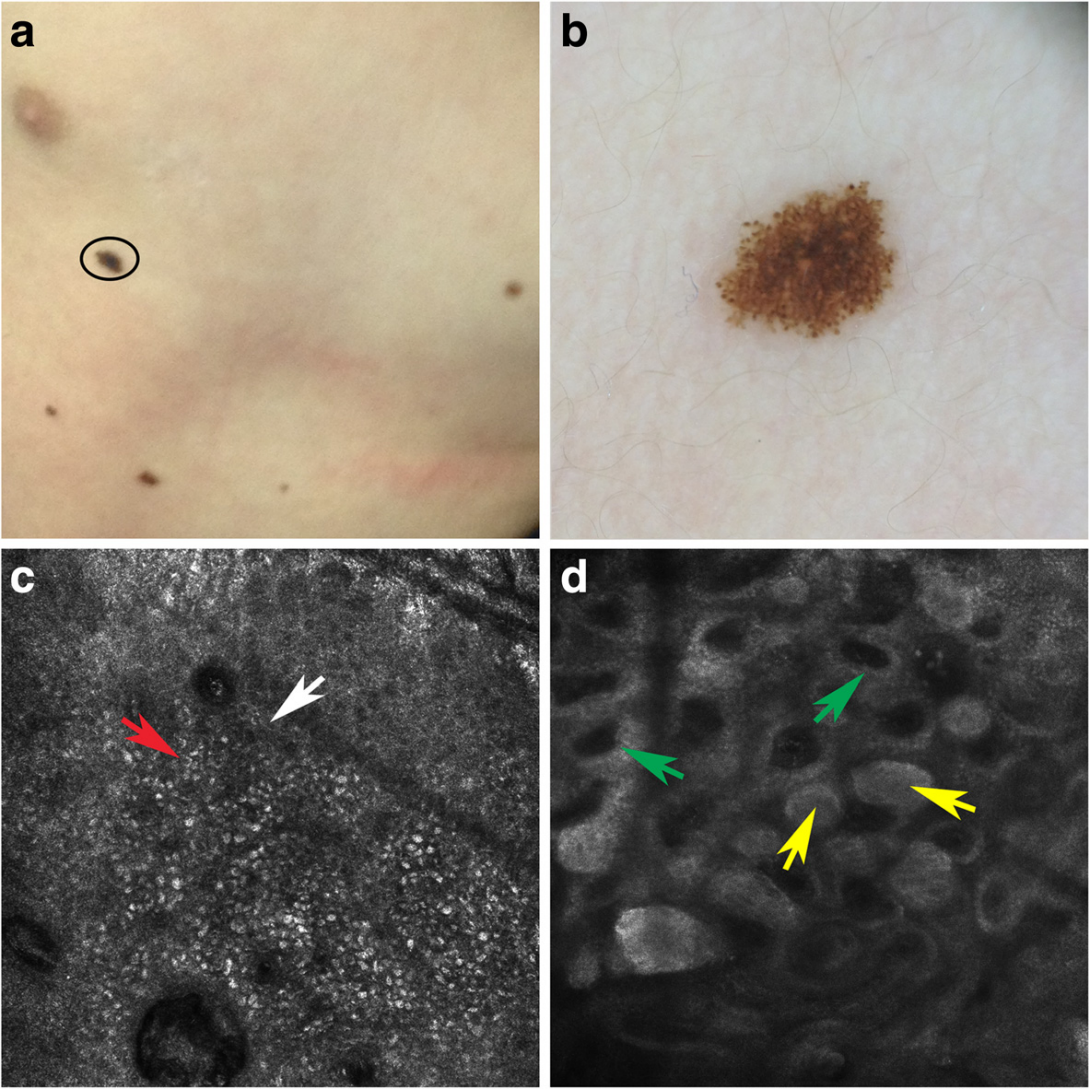
Spitz nevus localized on the trunk (**a**). Dermoscopy showed a globular pattern with regular, brownish central pigmentation and brownish globules at the periphery of the lesion (**b**). RCM revealed in the epidermis regular cobblestone with rare spindled (*white* arrow) and atypical cells (*red* arrow) (**c**). Instead at the dermoepidermal junction and within the papillary dermis showed the typical dense regular nests (*yellow* arrows). Moreover, papillary dermis presented edged papillae (*green* arrows) (**d**)

**Fig. 3:**
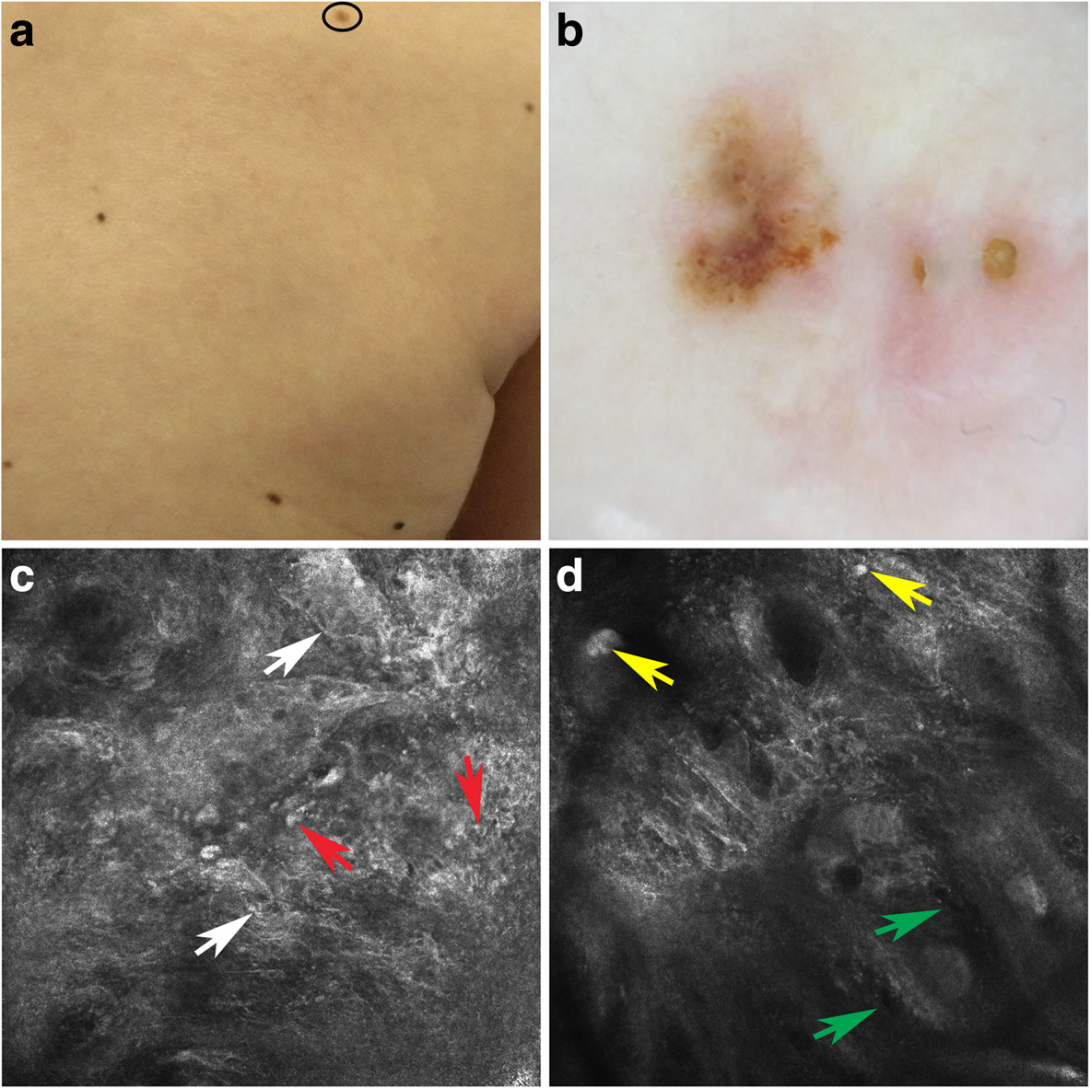
Spitz nevus localized on the back (**a**). Dermoscopy showed a multicomponent pattern with irregular pigmentation and a whitish-blue veil (**b**). RCM revealed in the epidermis irregular cobblestone with spindled (*white* arrows) and atypical cells (*red* arrows) (**c**). Instead at the dermoepidermal junction and within the papillary dermis showed nonedged papillae (*green* arrows) and plump bright cells (*yellow* arrows) (**d**)

A male neonate was born at 38 weeks’ gestation by vaginal delivery with vacuum extraction. Pregnancy was uneventful. Birth weight was 3290 g. The baby at birth was pale, hypotonic, bradycardiac, without spontaneous breathing and was promptly intubated. The Apgar score was 3, 6 and 6 at 1st, 5th and 10th minute respectively. Three hours after birth the neonate was referred to a neonatal intensive care unit. Due to intrapartum asphyxia the neonate was treated with hypothermia for 72 hours. Brain ultrasound scan (day 1) showed bilateral severe intraventricular hemorrhage (grade 3), while magnetic resonance imaging (day 4) revealed hydrocephalus with an extensive haematoma in the frontal lobes. The morphology of the bleeding was atypical, therefore a brain tumor was suspected to be the source of bleeding. An increasing ventricular dilatation was observed in the following days, therefore on day 7 haematoma was drained, an intraventricular catheter with an external shunt was placed and multiple biopsies were performed. The postoperative course was uneventful with the improvement of the hydrocefalus. Two days after surgery the baby was extubated and enteral feeding was started. Basing on the diagnosis of congenital GBM, confirmed by histology, on day 16 the external shunt was replaced with a reservoir (Ommaya). After positioning a central venous catheter (Broviac type monolumen) in the right atrium, chemotherapy was started at age 18 days and the patient was treated under the two-phase AIEOP Infant High Risk-CNS tumor protocol. Doses were adjusted for weight. The four-course Phase A included: methotrexate 250 mg/kg plus vincristine 0.04 mg/kg; etoposide 80 mg/kg; cyclophosphamide 135 mg/kg plus vincristine 0.04 mg/kg; carboplatin 25 mg/kg. Peripheral blood stem cells (PBSC) were collected for rescue therapy. Phase B included two high-dose chemotherapy regimens plus PBSC support: carboplatin 50 mg/kg plus etoposide 50 mg/kg; thiotepa 10 mg/kg/day plus melphalan 4 mg/kg/day. Common toxicity criteria (CTC) were adopted. Elevated transaminase levels (grade III) were observed after high dose methotrexate. Grade III anaemia and grade IV thrombocytopenia developed after the second (etoposide) and third (cyclophosphamide and vincristine) courses. Granulocyte colony stimulating factors (G-CSF) were given from day 35 to day 38. At the end of treatment hematopoietic stem cells were collected. On day 40 the reservoir was replaced with a ventricle-peritoneal drainage. The postoperative course was uneventful. A second curse of chemotherapy was started on day 48. After completion of treatment the clinical course was uneventful. To date no neurological impairments have been observed: development status, as assessed by motor, cognitive and social adaptive skills on the Clinical, Linguistic and Auditory Milestones (CLAM) scale, showed no significant differences from the normal range. The patient was born without any nevus clinically evident. From the age of 1 year some nevi appeared, mainly on his trunk and back. Because of the progressive increase in number and dimension of the pigmented skin lesions the child was sent to our attention for a dermatologic exam at the age of 16 months. The nevi had light-brown to black colour, with variable morphology and dimension from 2 mm to 21 mm, manly located on the trunk. The dermoscopic exam of these lesions revealed multiple atypical nevi characterized by a starburst pattern, globular pattern or multicomponent pattern [3, 4] with asymmetrical hypo-pigmented area and irregular borders. These dermatoscopic features correspond to Spitz nevi [3, 4]. To confirm our diagnostic suspicious we also performed the reflectance confocal microscopy (RCM) (VivaScope 1500®: Caliber I.D., Rochester, USA), which revealed in the nevi with starburst pattern (Fig. 1) and globular pattern (Fig. 2) the typical dense regular nests at the dermoepidermal junction and in the papillary dermis, while in the epidermis showed regular cobblestone with rare spindled and atypical cells [4]. Instead, the multicomponent pattern (Fig. 3) with RCM was characterized by irregular cobblestone with spindled and atypical cells in the epidermis, nonedged papillae and plump bright cells at the dermoepidermal junction and in the papillary dermis [4]. Therefore, we made a diagnosis of EDSN. We decided to perform a strict follow-up of the numerous Spitz nevi instead of performing multiple excisions. Nevertheless, during the follow-up we removed one lesion with multicomponent pattern (Fig. 3) suspected to be malignant which resulted to be a Spitz nevus at histopathological examination. Currently the patient is 3 years old and in the follow-up was not noted any changement in pattern and all nevi on dermoscopy were stable. In the follow-up carried out until now there has been observed the onset of new Spitz nevi.

## Discussion

Congenital GBM is a rare disease that accounts for only 3 % of congenital brain tumors and less than 30 cases have been reported in literature [1]. It is a very aggressive tumor with limited survival rates [1, 2]. The cause of the disease is unknown [2]. Patients with congenital GBM should undergo optimal surgical resection, radiation therapy and chemotherapy with different therapeutic approaches based on tumor extent and location [1, 2]. The Spitz nevus clinically appears as light-brown to black macule or papule, usually localized on the buttocks, arms or legs [3]. This nevus can reach size up to 5 mm in diameter [3, 4]. Spitz nevi are classified into 3 clinical variants: solitary, agminated and EDSN [3, 4]. The most common clinical variant is the solitary Spitz nevus, which occurs in children, and is usually localized on the neck or head [3, 4]. The agminated variant is characterized by Spitz nevi, which arise on congenital hypopigmented, and hyperpigmented patches. These lesions are commonly localized on the face and arms, with less frequent involvement of the thighs, neck, shoulders and other sites [5]. The EDSN is a rare clinical variant, which occurs predominantly in the adult, characterized by multiple Spitz nevi in the trunk, buttocks, elbows and knees [6]. The scalp, mucous membranes, palms and soles are usually spared. In the EDSN is possible to observe the development of more than 100 Spitz nevi, over a period of several months or years [6, 7]. To best of our knowledge only 15 cases of EDSN have been described in literature [7–9]. Generally, on dermoscopy the Spitz nevi shows three dermoscopic patterns: starburst, globular and multicomponent [3, 4]. Starburst pattern is characterised by multiple pigmented striations and/or large brown or black globules distributed symmetrically along the margins of the lesion, with a radiated appearance. Globular pattern is represented by regular brownish or greyish central pigmentation and brownish globules along the margins. Multicomponent pattern is characterized by irregular distribution of structures and colours, areas of diffuse, irregular pigmentation and a whitish-blue veil. RCM is a non-invasive imaging method for evaluating benign and malignant skin lesions and has a wide area of use in basic and clinical dermatology [10, 11]. RCM allows horizontal visualization of epidermis and superficial dermis with a nearly histologic resolution [4, 10, 11]. The typical aspects of Spitz nevi at RCM were previously described [4]. The cause of EDSN is still unknown, although a number of possible precipitating factors have been described in the literature, including pregnancy, intravenous drug abuse, perioperative stress, Addison disease, ultraviolet light exposure and fever following tonsillectomy [7, 12]. After chemotherapy a conspicuous eruption of multiple nevi has been reported in literature [13, 14]. Dysplastic nevi and malignant melanoma have been described in literature with concurrent or prior immunosuppressive states, such as in HIV infection, after renal transplantation and after chemotherapy, especially in children and young adults [13, 14]. In our case we hypothesize that EDSN is correlate with the chemotherapy. In detail we think that a plausible explanation of EDSN is the induction of nevocytic nevi by cytostatic agents. In fact cytostatic agents are mutagenic and can activate primordial nevus cell nests. To the best of our knowledge, there is no case of malignant transformation of Spitz nevi to melanoma in patients affected by EDSN [5–9, 12, 15]. Surgical removal of individual melanocytic lesions is possible but, due to the large number and poor cosmetic results, is not considered a good option for EDSN. Other treatment options adopted in the past are the liquid nitrogen, electrocoagulation and imiquimod [7, 15]. However, there is no satisfactory treatment for EDSN. Currently the best therapeutic choice is considered the clinical observation of melanocytic lesions.

## Conclusion

Patients with a history of multiple agent chemotherapy for treatment of pediatric malignancies have an increasing incidence of clinically and histologically multiple nevi, atypical nevi and malignant melanoma. We recommend a close follow-up of these patients with clinical observation, dermoscopy and RCM. However, we suggest the excision of lesions suspected of being malignant.

## Consent

Written informed consent was obtained from the patient’s legal guardian(s) for publication of this case report and any accompanying images. A copy of the written consent is available for review by the Editor-in-Chief of this journal.

## Abbreviations

GBM: glioblastoma multiforme
EDSN: eruptive disseminated Spitz nevi
RCM: reflectance confocal microscopy
PBSC: peripheral blood stem cells
CTC: common toxicity criteria
CLAM scale: Clinical, Linguistic and Auditory Milestones scale
G-CSF: granulocyte colony stimulating factors
